# The impact of an antimicrobial stewardship program restriction on remdesivir prescribing

**DOI:** 10.1017/ash.2022.290

**Published:** 2022-08-31

**Authors:** Kristen E. Zeitler, Nicholas Piccicacco, Ripal Jariwala, Jose Montero, Kami Kim

**Affiliations:** 1 Department of Pharmacy, Tampa General Hospital, Tampa, Florida; 2 Department of Pharmaceutical Services, University of California San Francisco, San Francisco, California; 3 Division of Infectious Diseases, Department of Internal Medicine, University of South Florida Morsani College of Medicine, Tampa, Florida; 4 Global Emerging Diseases Institute, Tampa General Hospital, Tampa, Florida

*To the Editor—*Remdesivir, an intravenous direct-acting nucleotide inhibitor with in vitro activity against severe acute respiratory syndrome coronavirus 2 (SARS-CoV-2), was the first treatment to receive full Food and Drug Administration (FDA) approval for coronavirus disease 2019 (COVID-19) in October 2020.^1^ Prior to this approval, remdesivir was also the first COVID-19 therapeutic to receive emergency use authorization (EUA) from FDA. Due to its initial limited supply, remdesivir use was restricted at our institution to the antimicrobial stewardship program to allocate supply to appropriate patients.

**Figure 1.**
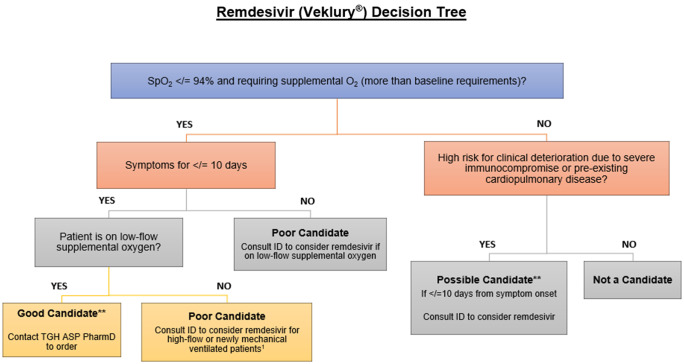
Institutional Remdesivir Ordering Restriction Algorithm.

At Tampa General Hospital (TGH), a 1,041-bed academic medical center affiliated with the University of South Florida Morsani College of Medicine, >7,700 patients have been admitted due to COVID-19 as of July 22, 2022. The TGH antimicrobial stewardship (ASP) team, consisting of 3 infectious diseases (ID)–trained pharmacists and an ID-trained physician, developed and maintained evidence-based institutional guidelines for the appropriate use of SARS-CoV-2–directed therapeutics agents beginning in March 2020. Additional stakeholders within the ID division and throughout the institution supported and followed these guidelines. Based on data from randomized controlled trials conducted by Beigel et al^2^ and Goldman et al,^3^ our team created a remdesivir treatment algorithm for providers (Fig. 1) as well as a hospital-wide remdesivir ordering restriction in the electronic medical record (EMR) allowing therapy to be utilized in patients within 10 days from symptom onset and in those not on invasive mechanical ventilation. If therapy was appropriate, medication ordering in the EMR was limited to the ASP team; all other individuals attempting to order remdesivir in the EMR were presented with a message indicating the restriction required direct discussion with the ASP team. If the patient did not meet criteria, the ASP pharmacist would provide education and/or recommend an ID consultation to weigh the risks and benefits of starting remdesivir. This restriction was in place 24 hours a day, 7 days a week. Due to the impact of the COVID-19 pandemic on our ID service line, ID fellows were not involved in this workflow. This hard-stop ordering restriction continued until September 2021. At that time, by consensus, a culture requirement was established regarding remdesivir use. EMR ordering changed to a soft stop with order questions that required providers to document their patient qualified for therapy per institutional guidance.

To evaluate the impact of our remdesivir restriction, we compared our utilization of remdesivir from November 2020 through January 2022 to similar institutions in the Vizient Clinical Data Base (Irving, TX), the largest member-driven, healthcare performance improvement company in the United States. This comparator hospital group included 40 institutions with ≥ 750 beds, a Medicare case-mix index ≥2.0, ≥15,000 annual discharges, and ≥10,000 annual inpatient surgeries. Overall remdesivir utilization data were determined by generating a resource utilization report by hospital/hospital system and querying for the following terms: “COVID-19” as any diagnosis code, “remdesivir,” “inpatient,” “November 2020 through January 2022,” and “TAMPAGEN 100128.”^4^ This time frame was used for the analysis based on FDA approval for remdesivir in October 2020. Thereafter, the drug was acquired by direct purchase, but prior to this, the drug was available free of charge under EUA status. Our institution utilized remdesivir in 28.2% of all inpatients with a diagnosis of COVID-19, for a mean of 4.2 days per case. This metric compares to the overall population of similar institutions where utilization ranged from 17.4% to 66.5% (median, 44.7%), and the mean days of remdesivir use ranged from 3.5 to 4.8 days. In this comparison group, our institution ranks in the 15th percentile in remdesivir use during the targeted time frame.

Utilizing this data, a remdesivir cost mitigation analysis was conducted based on use of remdesivir in 28.2% of patients at our institution during the prespecified period from November 2020 through January 2022 (4,277 total patients). For this analysis, we used a remdesivir acquisition cost of $520 per 100-mg vial and a total cost of $3,120 for a 5-day course (200 mg on day 1, followed by 100 mg on days 2–5). We also used the median percentage use of remdesivir from our comparator hospital group of 44.7%. Extrapolating these data, we estimated an avoidance of 706 courses of remdesivir (5-day duration of therapy). This would create a $2.2 million cost savings at our institution due to the ordering restriction.

We acknowledge the assumptions made with these data, namely that institutions approached the use of remdesivir differently and that data during the COVID-19 pandemic were changing rapidly. We did not have data specific to volumes of patients during SARS-CoV-2 variant surges (ie, δ [delta] and ο [omicron] variants) experienced at comparator institutions. However, we suspect that each institution was similarly affected by the variant waves based on national data and the types of institutions we included in our comparator analysis. Our use of an ASP-based restriction pathway ensured that the latest evidence-based literature was utilized, although we recognize that this approach affected the ASP team’s focus on other daily stewardship activities. This initiative was successful due to the strong collaboration between our ASP team, ID providers, engaged medical staff, hospital leadership, and all those who cared for COVID-19 patients. In conclusion, our institutional data show that an ASP team can work with providers to create a concise COVID-19 treatment guideline that yields direct cost savings.
